# The effect of calcium hydroxide on the steroid component of Ledermix® and Odontopaste®

**DOI:** 10.1111/j.1365-2591.2011.01940.x

**Published:** 2011-12

**Authors:** M Athanassiadis, N Jacobsen, P Parashos

**Affiliations:** 1 Australian Dental ManufacturingBrisbane, Qld.; 2 Dental School, University of MelbourneMelbourne, Vic., Australia

**Keywords:** calcium hydroxide, Ledermix Paste, Odontopaste, triamcinolone acetonide

## Abstract

**Aim:**

To investigate the chemical interaction of calcium hydroxide with the corticosteroid triamcinolone acetonide in Ledermix® Paste and in Odontopaste®, a new steroid/antibiotic paste.

**Methodology:**

Validated methods were developed to analyse the interaction of calcium hydroxide in two forms, Pulpdent® Paste and calcium hydroxide powder, with triamcinolone acetonide within Odontopaste® and Ledermix® Paste. High-performance liquid chromatography (HPLC) was used to analyse the mixed samples of the pastes and calcium hydroxide. The concentration of triamcinolone acetonide within the pastes was determined over 0, 2, 6, 24 and 72-h time-points. All tests with the HPLC involved the testing of the standard with triplicate injections alongside the samples. All samples were tested in duplicate with each injected twice; therefore, four tests were performed for each investigation. Linearity, precision and specificity of the testing procedures and apparatus were validated. Descriptive statistics are provided.

**Results:**

In both pastes, there was a marked rapid destruction of the triamcinolone acetonide steroid upon mixing with calcium hydroxide. Odontopaste® suffered a lower rate of destruction of the triamcinolone acetonide component than Ledermix® Paste, but both pastes showed very similar degrees of steroid destruction after 72 h. When using calcium hydroxide powder with Ledermix® Paste, the triamcinolone was destroyed entirely and immediately.

**Conclusion:**

The addition of calcium hydroxide to Odontopaste® or Ledermix® Paste results in the rapid destruction of the steroid.

## Introduction

Calcium hydroxide paste is a root canal medicament that has been used in endodontics for many years. It exhibits antibacterial properties and has the ability to stimulate hard tissue repair within bone and cementum (Heithersay 1975, Tronstad *et al.* 1981). Ledermix® Paste (Haupt Pharma GmbH, Wolfratshausen, Germany) has been recommended for endodontic use owing to its anti-inflammatory effect (Ehrmann 1981). Odontopaste® (Australian Dental Manufacturing, Kenmore Hills, Qld, Australia) has recently been released onto the dental market, similarly for its anti-inflammatory effect. The main difference between Odontopaste® and Ledermix® Paste is that clindamycin hydrochloride in Odontopaste® replaces demeclocycline hydrochloride in Ledermix® Paste. Clindamycin hydrochloride has an equivalent spectrum of antibacterial activity but exhibits minimal staining of teeth (Adamidou 2010).

Calcium hydroxide and the two steroid/antibiotic pastes serve different purposes, so in an effort to obtain the benefits of both, a 50 : 50 mix of calcium hydroxide and Ledermix Paste has been recommended (Abbott *et al.* 1989, Taylor *et al.* 1989). This early research was based on the use of tritium-labelled triamcinolone acetonide and liquid scintillation counters to detect the beta decay of tritium, which correlates the counts with a concentration of the tritium-labelled component (Abbott *et al.* 1989). However, the exact nature of the molecule labelled by the tritium is not known because there are a possible 31 positions for the tritium-labelled hydrogen on a molecule of triamcinolone acetonide, and previous research has not identified whether the scintillation counter was measuring the labelled steroid or a breakdown product. Triamcinolone acetonide is also polymorphic. British Pharmacopoeia (BP) and United States Pharmacopeia (USP) grades of triamcinolone acetonide conform to a particular type of the possible polymorphic forms, and the manufacturing process can control which polymorphic form is produced. Whether this remains the case with the tritium-labelled triamcinolone acetonide is unknown. Previous studies have found varying degrees of breakdown of steroids at high pH (Dekker & Buijs 1980, Hansen & Bundgaard 1980, Ogata *et al.* 1998, Spangler & Mularz 2001, Conrow *et al.* 2002, Teng *et al.* 2003, Nygaard *et al.* 2004, Görög 2010), so it is conceivable that the tritium labelling may not be on an active molecule, but may be on a breakdown product.

Therefore, this study was undertaken to investigate the effects of the addition of calcium hydroxide in the form of calcium hydroxide powder and in the form of Pulpdent® Paste (Pulpdent Corporation, Watertown, MA, USA) on the stability of the steroid component of two endodontic pastes, Odontopaste® (Australian Dental Manufacturing) and Ledermix® (Haupt Pharma GmbH).

## Materials and methods

The two pastes along with the other products utilized in this research project are summarized in Table 1. All tests were carried out independently by an external laboratory licensed by the Australian Therapeutic Goods Administration (TGA) and with Good Laboratory Practice certification. All batches were tested in compliance with manufacturers’ claims prior to testing.

**Table 1:** A comparison of the materials used and their respective ingredients

Ingredient (%w/w)	Odontopaste®	Ledermix®	Pulpdent®	Calcium hydroxide powder BP grade
Clindamycin hydrochloride	5%	–	–	–
Demeclocycline hydrochloride (calcium salt)	–	3%	–	–
Triamcinolone acetonide	1%	1%	–	–
Base paste incipient ingredients	Equivalent	Equivalent	–	–
Purified water	Present	Present	Present	–
Calcium hydroxide	Present	–	40%	>98%
Methyl cellulose	–	–	Present	–

BP, British pharmacopoeia.

### Method validation for Triamcinolone Acetonide in Odontopaste®/Ledermix®

The method for detecting triamcinolone acetonide was validated for each paste. High-performance liquid chromatography (HPLC) was used to analyse the mixed samples of paste and calcium hydroxide. The validation parameters that were measured and the assessment of the results were chosen so as to conform to the following protocols:

- The International Conference on Harmonisation of Technical Requirements for Registration of Pharmaceuticals for Human Use (ICH) – Validation of Analytical Procedures: Text and Methodology as adopted by the TGA, Australia (European Medicines Agency 2006),
- Guidelines for the Validation and Verification of Chemical Test Methods. National Association of Testing Authorities, NATA Technical Note 17 (NATA 2009).
- Guidelines for the Validation of Analytical Methods for Active Constituents, Agricultural and Veterinary Chemical Products October 2004 (APVMA 2004),

Where no acceptance criteria existed other recognized sources were used, predominantly, the Association of Analytical Communities-Single Laboratory Validation Acceptance Criteria (AOAC 2009).

For all analyses, all pH readings were taken utilizing a Mettler Toledo 7s calibrated pH metre (Mettler Toledo, Aurora, IL, USA). A Varian ProStar (Agilent Technologies, Palo Alto, CA, USA) with ternary gradient pump and programmable single channel UV detector was used. The columns consisted of Allsphere ODS-2 (Allsphere, Woodstock, IL, USA), 5 μm, 4.6 × 250 mm. The column guards were Phenomenex Security Guard C18 inserts (Torrance, CA, USA). The data were collected using Varian Star software. The reference standard for triamcinolone acetonide was obtained from Sigma-Aldrich (St. Louis, MO, USA). The reagents used were a mixture of methanol (HPLC grade), water and glacial acetic acid. Operating conditions were as follows: flow rate was 1.2 mL min^−1^, equilibrium time was ten minutes, detector wavelength was 283 nm, the injection volume was 20 μL and the retention time was approximately 4.6 min. Standards were prepared by weighing 10 and 100 mg of triamcinolone acetonide (taking into account the potency of the steroid) into 100 mL of methanol. The flask was ultrasonicated until the triamcinolone acetonide had fully dissolved. The solution was then filtered through 0.45 μm filter paper into the HPLC vial. The samples were prepared by taking the well-mixed paste, which theoretically contained 10 and 100 mg of triamcinolone acetonide, and this was added to the solution as above and filtered with 0.45 μm filter paper into the HPLC vial.

Samples were run through in duplicate, and the calculations were made according to the following formula: 
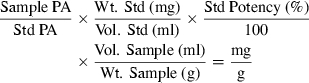
 where PA = peak area.

**Figure 1:**
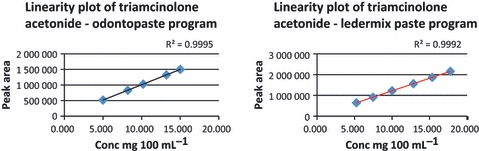
Linear calibration curve for method validation. A determination for the standard deviation was made.

Linearity (i.e. the ability within a given range to obtain test results that are directly proportional to the concentration of the analyte in the sample being tested) was validated using five concentration standards of triamcinolone acetonide (Sigma-Aldrich) with a potency of 99.5%. These standards (Table 2) were analysed, and the resultant areas were used to prepare a calibration curve (Fig. 1) that was then used to determine the measured concentration of each of the five calibration standards.

**Table 2:** Concentration of standards used for validation of linearity. The measured concentrations are compared to the actual

Triamcinolone acetonide added (mg)	Concentration mg per 100 mL	Average peak area	Measured concentration mg per 100 mL
Odontopaste® testing program (Potency of the standard is 99.5%)			
15.2	15.124	1 499 099	15.017
13.2	13.134	1 322 166	13.215
10.2	10.149	1 026 276	10.203
8.2	8.159	830 246	8.207
5.1	5.075	515 201	4.999
Ledermix® Paste testing program (Potency of the standard is 99.4%)			
17.93	17.826	2 158 333	17.78
15.37	15.279	1 866 610	15.36
12.81	12.733	1 562 085	12.85
10.25	10.186	1 223 774	10.01
7.69	7.640	910 983	7.47
5.12	5.093	642 576	5.25

Precision of the testing apparatus was considered at three levels, repeatability, intermediate precision and reproducibility. System precision for Odontopaste® utilized 10.2 mg per 100 mL triamcinolone acetonide at 99.5% potency. The standard was injected six times, as required by the International Conference on Harmonisation (European Medicines Agency 2006). System precision for Ledermix® Paste utilized 10.2 mg per 100 mL triamcinolone acetonide at 99.4% potency. The standard was injected six times.

The AOAC (2009) considers that for samples containing 0.01% of the analyte, a repeatability of 4% is recommended. Repeatability is measured by the relative standard deviation (RSD), and as such, the RSD of 0.17% for Odontopaste® and 1% for Ledermix® Paste is considered acceptable.

Specificity (to validate that the analyte alone was being tested, free from the effects of the matrix of other ingredients) was performed by analysing a placebo that is a sample containing all of the ingredients except the active, and the analysis of the placebo spiked with the analyte. Whilst the placebo could be considered as a negative control, in this type of analysis, it is utilized to determine peak purity rather than validating a method. A placebo was prepared by ADM of Odontopaste® and tested. Approximately 1 g of the placebo mix was subsampled and extracted as per the method originally developed. An additional 1 g placebo was then subsampled, and a known amount of triamcinolone acetonide was added and then extracted according to the method. This formed the spiked sample. In addition, spiked samples were also tested for specificity. Interference from the placebo was not observed, and the spike recovery of 97.6% is within the limits of 85–110% as recommended by the AOAC (2009). As such, the specificity of the method was considered acceptable.

For Ledermix® Paste, it was decided to not produce a placebo as there may have been questions raised concerning the accuracy of the placebo's formulation. Specifically, because the exact formula of Ledermix® Paste is ‘commercial-in-confidence’, attempting to produce an accurate placebo would not have been possible. It was therefore decided to extract the triamcinolone acetonide from a sample and a spiked sample (i.e. 30% spike on half the sample weight so that it remained within the linear range of the validation). This was then analysed. The results were within the limits recommended by the AOAC (2009), and hence, specificity was considered acceptable.

Accuracy for Odontopaste® was investigated by the analysis of a number of placebo subsamples spiked with triamcinolone acetonide. Six separated determinations were performed. Approximately 1.0 g of placebo was subsampled into six separate vessels. The extraction of triamcinolone acetonide was carried out as per the method earlier. The average recovery was 97.6%, which was again within the accepted range (AOAC 2009), and considered acceptable. The accuracy testing for Ledermix® Paste was investigated by analysing a number of samples spiked with different levels of the triamcinolone acetonide standard. Nine separate determinations were performed, incorporating three replicate spikes at three different concentration levels of 8 mg per 100 mL, 10 mg per 100 mL and 13 mg per 100 mL as described in the accuracy-spiking recovery data. Duplicate injections of each spike were carried out.

A stock solution of triamcinolone acetonide standard was prepared (104.61 mg per 100 mL triamcinolone acetonide – potency 99.4%). Samples containing approximately 50 ppm active were spiked at 3 mg per 100 mL, 5 mg per 100 mL and 8 mg per 100 mL of triamcinolone acetonide standard and extracted. Each spiked level was performed in triplicate. The levels of spike were chosen so that the total concentrations of active in the spikes were within the linear range of this validation. The sample and spikes were analysed, and the recoveries were determined individually. The per cent recovery for the three levels of spikes meets the acceptance criteria for a 1.0% concentration solution of 92–105% for triamcinolone acetonide.

### Testing of starting materials

All tests with the HPLC involved the testing of the standard with triplicate injections alongside the samples. Results were rejected from the test run if the differences between the standard's results were >3%. All test samples were tested in duplicate with each injected twice; therefore, four tests were performed for each investigation. If the results for the samples tested for each individual investigation differed by more than 3%, they were rejected. Throughout the testing, no rejections occurred.

Odontopaste® and Ledermix® pastes were tested for the initial concentration of triamcinolone acetonide. Pulpdent® was tested for the percentage of calcium hydroxide present. The calcium hydroxide raw powder was of BP grade and was tested for purity.

### Testing of combinations

The following products were mixed in a 50 : 50 ratio by weight:

- Time-point analysis
- Ledermix® Paste with Pulpdent®
- Ledermix® Paste with Calcium Hydroxide Powder BP grade
- Odontopaste® with Pulpdent®
- Odontopaste® with Calcium Hydroxide Powder BP grade
- Analysis at zero time-point only
- Pulpdent® with a 1.4% triamcinolone acetonide only paste in an aqueous paste
- Pulpdent® with a 1.4% triamcinolone acetonide only paste in a peg/aqueous paste

The following was also tested for concentrations of triamcinolone acetonide:

- Odontopaste® with 2% calcium hydroxide w/w
- Odontopaste® with 0.5% calcium hydroxide w/w
- Calcium hydroxide powder with 2% triamcinolone acetonide w/w

For the time-point analyses, the ingredients were mixed in a closed container for 3 min. For a large volume of paste, there was a concern that the mixing would not result in a homogeneous paste particularly if clinical times of <30 s were used. Therefore, it was decided to mix for longer than what would be performed so clinically. Following the mixing, the samples were then subsampled from the closed container and placed through the HPLC to determine the amount of triamcinolone acetonide present at differing time-points. The time taken for the samples to be analysed was approximately 3 min. They were then tested for the presence of triamcinolone acetonide at 0, 6, 24 and 72 h.

The Pulpdent® samples that were mixed 50 : 50 with custom-made 1.4% triamcinolone acetonide pastes were tested at zero time-point only. The two custom samples were produced to eliminate many of the Odontopaste® and Ledermix® Paste ingredients, thereby narrowing down the possible cause of the steroid's degradation.

The two samples of Odontopaste® with 0.5% calcium hydroxide and 2% calcium hydroxide were prepared under ISO 13485:2003, a medical device quality assurance system, and tested prior to use. The mixing was validated to ensure no issue with the homogeneity of the paste.

Because this work did not aim to compare the two products, only descriptive statistics were originally intended, but the appropriateness of statistical analysis was to be based on the raw data.

## Results

The initial testing of the ingredients resulted in the concentrations of the key components as listed in Tables 3–5. The time-point testing results for the percentage of triamcinolone acetonide when Odontopaste® and Ledermix® were mixed with calcium hydroxide powder BP grade are listed in Table 6 demonstrating rapid loss of the steroid. Similar results were obtained with Pulpdent® paste, although the initial and overall destruction of the steroid in Odontopaste was less (Table 7). Rapid and extensive destruction by Pulpdent® Paste of the 1.4% triamcinolone acetonide occurred also in the custom-made pastes (Table 8). Increasing concentrations of calcium hydroxide demonstrated increasingly destructive effect of the steroid (Table 9). Because the degree of destruction of the steroid component of both pastes was so similar, the need for statistical comparison was considered superfluous.

**Table 3:** The percentage of triamcinolone acetonide and the pH of Odontopaste® and Ledermix® Pastes prior to mixing with calcium hydroxide

Product	% of triamcinolone acetonide prior to testing	pH value
Odontopaste®	1.14^a^	8.5
Ledermix® Paste	1.0	8.2

a The use of overage accounts for the higher initial concentration.

**Table 4:** The percentage of calcium hydroxide in Pulpdent® and the BP grade of calcium hydroxide powder used prior to the mixing of the components

Product	% (w/w) of calcium hydroxide	pH value
Pulpdent®	19.2	12.5
Calcium hydroxide powder BP grade	98.2	–

BP, British pharmacopoeia.

**Table 5:** Initial testing of custom-made triamcinolone acetonide pastes to validate the presence of steroid

Product	% (w/w) of triamcinolone acetonide
Custom-made triamcinolone acetonide paste in aqueous only paste	1.4
Custom-made triamcinolone acetonide paste in peg/aqueous paste	1.4

**Table 6:** The time-point results for triamcinolone acetonide when powdered calcium hydroxide with 98.2% purity is mixed with Odontopaste® and Ledermix® in a 50 : 50 ratio

Compound	Premixing (%)	0 h (%)	1 h (%)	6 h (%)	24 h (%)	72 h (%)
Odontopaste®	1.14	0.09	0.07	0.03	0.01	0.03
		−84	−87	−94	−98	−94
Ledermix®	1.00	0	0	0	0	0
		−100	−100	−100	−100	−100

Percentage loss was calculated on 50% of premix percentages owing to dilution with the 50 : 50 mixing. e.g. −84% = (0.09%/(1.14%/2)) − 100%.

**Table 7:** The time-point results for triamcinolone acetonide when Pulpdent® is mixed with Odontopaste® and Ledermix® in a 50 : 50 ratio

Compound	Premixing (%)	0 h (%)	1 h (%)	6 h (%)	24 h (%)	72 h (%)
Odontopaste®	1.14	0.440	0.310	0.200	0.350	0.060
		−20	−44	−64	−36	−89
Ledermix®	1.0	0.097	0.079	0.063	0.084	0.020
		−81	−84	−87	−83	−96

Percentage loss was calculated on 50% of premix percentages owing to dilution with the 50 : 50 mixing.

**Table 8:** The results for the addition of Pulpdent® 50 : 50 with custom-made triamcinolone acetonide pastes

Compound	Premixing (%)	Post mixing (%)
1.4% Triamcinolone acetonide in aqueous paste mixed with Pulpdent® 50 : 50	1.4	0.25
1.4% Triamcinolone acetonide in aqueous/peg paste mixed with Pulpdent® 50 : 50	1.4	0.16

**Table 9:** The effect on the triamcinolone acetonide component of Odontopaste® with different amounts of calcium hydroxide and the effect of dry calcium hydroxide powder when mixed with triamcinolone acetonide

Compound	Amount of calcium hydroxide (%)	Triamcinolone acetonide added (%)	Triamcinolone acetonide recovered (%)
Odontopaste®	0.5	1.14	1.14
Odontopaste®	2	1.14	0.53
Calcium hydroxide powder	98	2	0

## Discussion

The initial testing of all the components revealed that Pulpdent^**®**^ Paste contained 19.2% calcium hydroxide. This was markedly and unexplainably lower than the manufacturer's specification of 40%. However, it was decided to continue the testing with this lower amount as this may reflect the true clinical concentration of calcium hydroxide paste when stored and used in a clinical environment. With less calcium hydroxide, the effects were expected to be milder than if the Pulpdent^**®**^ Paste had calcium hydroxide present at 40%. All the other utilized products tested were as per the manufacturers’ specification. The higher initial amount of triamcinolone acetonide in Odontopaste^**®**^ in comparison with Ledermix^**®**^ was owing to the use of ‘overage’ in its manufacture. It is not uncommon for manufacturers to compensate for loss of actives during the manufacturing process or to compensate for lack of purity of the active ingredients. This is referred to as ‘overage’ and is a percentage of the active ingredient above the labelled amount. Odontopaste^**®**^ uses the same process of overage for its manufacture. Hence, the labelled amount on the packaging, in general, refers to the amount present at the time of expiry, and the product may contain higher amounts of the active ingredients at the time of initial manufacture to compensate for the loss of the same actives over time. As a result, the inactive ingredients, more commonly referred to as excipients, may vary from batch to batch depending on the overage adjustments required.

The effect of calcium hydroxide on the steroid component, triamcinolone acetonide, of both Odontopaste® and Ledermix® Pastes was considerable. It resulted in a reduction in the triamcinolone acetonide component in both Odontopaste® and Ledermix®. There was less loss of the steroid component in Odontopaste® in both the calcium hydroxide powder and Pulpdent® Paste groups. The 24-h time-point results showed an increase in the amount of triamcinolone acetonide, and this can only be explained by the mixing not being homogeneous. These results were not discarded and are valuable in that they show the effect that mixing may have on the interaction of calcium hydroxide with triamcinolone acetonide. In clinical situations, the mixing is commonly performed within the canal because the consistency after mixing is such that attempting to place the mixture with a lentulo-spiral bur or paste-filler bur is very difficult and inefficient.

In addition, the level at which the steroid, triamcinolone acetonide, is therapeutic within a root canal is unknown. Therefore, whether or not there is any loss of the therapeutic effect from the reduced levels of the steroid cannot be definitively determined. A recent study concluded that a level >1% triamcinolone acetonide was preferred or that stronger steroids such as dexamethasone or prednisone be utilized (Chen *et al.* 2008). Therefore, any loss of steroid should be of concern.

It is clear that triamcinolone acetonide was actively broken down by calcium hydroxide. The tests with the custom pastes in Table 8 confirm that no other ingredients are responsible for the destruction of the triamcinolone acetonide other than the calcium hydroxide. Interestingly, the addition of triamcinolone acetonide to raw calcium hydroxide powder also resulted in the rapid destruction of the triamcinolone acetonide (Table 9). The chemistry behind the reaction is not fully understood. It is unlikely that degradation occurs when the products are in powdered form. For the breakdown to occur, an aqueous solution is required. However, when calcium hydroxide is exposed to carbon dioxide in the atmosphere, it converts to calcium carbonate with the concomitant production of water (Armstrong 2009). This water may provide a sufficient vehicle for the activity of the hydroxyl ions to destroy the steroid. However, the amount of water produced from this reaction is small, reducing the likeliness of this scenario. A more probable explanation may be the presence of an aqueous component from the extraction media used when injecting the test materials into the HPLC. The aqueous component may provide the environment required for the dissociation of the calcium hydroxide in the test sample and therefore the formation of the hydroxyl ions, which degrade the steroid in an oxidative reaction (Hansen & Bundgaard 1980). This is a more plausible explanation and would reinforce the rapid rate of degradation of triamcinolone acetonide found in the presence of calcium hydroxide.

These results indicate that there is no real advantage with mixing calcium hydroxide with Ledermix® or Odontopaste®. Furthermore, there is no significant antibacterial advantage of the combinations when compared to calcium hydroxide alone (Plutzer 2009). In addition, what occurs to the antibiotic by the calcium hydroxide has not been investigated. Another important consideration is the known issue of staining of teeth with Ledermix® (Kim *et al.* 2000, Adamidou 2010, Day *et al.* 2011). Staining is not clinically significant with Odontopaste® and most calcium hydroxide formulations (Adamidou 2010). Hence, if a purely antibacterial action is required, then the use of calcium hydroxide is sufficient. If a steroid action is required, then the use of Odontopaste® or Ledermix® would seem acceptable with only Odontopaste® not causing the teeth to stain.

As a corollary to the results of this study, a lower level of calcium hydroxide may be satisfactory as an antibacterial agent. There have been no studies to accurately indicate what percentage of calcium hydroxide is sufficient to provide an antibacterial effect within a root canal. The 50 : 50 mixing may indicate that 20% Pulpdent® is just as effective as 40% Pulpdent® giving some insight into the therapeutic threshold for calcium hydroxide as an antibacterial.

## Conclusions

The results of this study do not validate the previous research regarding the addition of calcium hydroxide to Ledermix® Paste. They do however validate research performed on the breakdown of steroids in an alkaline environment. The addition of calcium hydroxide in the form of Pulpdent® or BP grade calcium hydroxide powder to Odontopaste® or Ledermix® Paste in a 50 : 50 ratio results in the rapid destruction of the steroid, triamcinolone acetonide. Calcium hydroxide, therefore, cannot reduce the rate of diffusion of all the active components within the paste and would not make the medicament last longer within the canal. It does not assist in keeping a higher concentration of all the components within the canal. The validity and continued use of calcium hydroxide with Odontopaste® and Ledermix® Pastes should be reconsidered, and further testing is required to determine the breakdown products produced.
